# Copper Deficiency Leads to Anemia, Duodenal Hypoxia, Upregulation of HIF-2α and Altered Expression of Iron Absorption Genes in Mice

**DOI:** 10.1371/journal.pone.0059538

**Published:** 2013-03-28

**Authors:** Pavle Matak, Sara Zumerle, Maria Mastrogiannaki, Souleiman El Balkhi, Stephanie Delga, Jacques R. R. Mathieu, François Canonne-Hergaux, Joel Poupon, Paul A. Sharp, Sophie Vaulont, Carole Peyssonnaux

**Affiliations:** 1 INSERM, U1016, Institut Cochin, Paris, France; 2 CNRS, UMR8104, Paris, France; 3 Université Paris Descartes, Sorbonne Paris Cité, Paris, France; 4 Laboratoire de Toxicologie Biologique, Hôpital Lariboisière, Paris, France; 5 INSERM U1043-CPTP, Toulouse, France; 6 CNRS, U5282, Toulouse, France; 7 Université de Toulouse, UPS, Centre de Physiopathologie de Toulouse Purpan (CPTP), Toulouse, France; 8 King’s College London, Diabetes & Nutritional Sciences Division, London, United Kingdom; 9 Department of Pharmacology and Cancer Biology, Duke University, Duke University Medical Center, Durham, North Carolina, United States of America; Queen Mary University of London, United Kingdom

## Abstract

Iron and copper are essential trace metals, actively absorbed from the proximal gut in a regulated fashion. Depletion of either metal can lead to anemia. In the gut, copper deficiency can affect iron absorption through modulating the activity of hephaestin - a multi-copper oxidase required for optimal iron export from enterocytes. How systemic copper status regulates iron absorption is unknown. Mice were subjected to a nutritional copper deficiency-induced anemia regime from birth and injected with copper sulphate intraperitoneally to correct the anemia. Copper deficiency resulted in anemia, increased duodenal hypoxia and Hypoxia inducible factor 2α (HIF-2α) levels, a regulator of iron absorption. HIF-2α upregulation in copper deficiency appeared to be independent of duodenal iron or copper levels and correlated with the expression of iron transporters (Ferroportin - Fpn, Divalent Metal transporter – Dmt1) and ferric reductase – Dcytb. Alleviation of copper-dependent anemia with intraperitoneal copper injection resulted in down regulation of HIF-2α-regulated iron absorption genes in the gut. Our work identifies HIF-2α as an important regulator of iron transport machinery in copper deficiency.

## Introduction

Copper and iron are essential trace metals required for many biochemical processes, however, their redox properties also make the free metal potentially toxic. As a result, uptake and intracellular distribution of both metals are tightly regulated. Both iron and copper are absorbed from diet across the duodenal brush border membrane by their respective carriers – Divalent metal-ion transporter 1 (DMT1) and Copper transporter 1 (CTR1). This transport step is preceded by the reduction of either iron or copper to their ferrous (Fe^2+^) or cuprous (Cu^+^) forms, respectively. The reduction of iron is mediated by brush border ferric reductases including duodenal cytochrome b (DCYTB) ferric reductase [1]. The nature of the intestinal cupric reductase is less clear. Both DCYTB and STEAP proteins have been put forward as candidates [2], [3]. At the basolateral membrane, ferroportin (FPN) and ATP7A mediate iron and copper export, respectively. In addition, a multi-copper oxidase, hephaestin (HEPH), is required for optimal FPN-mediated iron export, thus linking intestinal copper and iron homeostasis - for review see [4].

The regulation of components of the iron absorption pathway occurs at both the transcriptional and post-transcriptional levels. HIF-1 and HIF-2 are heterodimeric transcriptional factors (TFs) and central mediators of cellular and systemic adaptation to hypoxia. In the presence of oxygen, the HIF-α subunit is hydroxylated by oxygen, 2-oxoglutarate (co-substrates) and iron (co-factor) dependent prolyl hydroxylases (PHDs) and targeted to the proteasome after binding to the von Hippel-Lindau (vHL) protein. In hypoxia, HIF-α is stabilized and binds to the HIF-β constitutive subunit to promote transcription of target genes [5]. We and others have shown that HIF α directly regulates the expression of iron absorption-related genes: *Slc11a2* (*1a* promoter*)*, *Slc40a1 (1b* promoter) and *Cybrd1* which code for DMT1, FPN and DCYTB respectively. This regulation occurs at both the basal level [6], [7] and in the context of the adaptive response to iron deficiency [8].

In addition, enterocyte iron homeostasis is post-transcriptionally coordinated by RNA binding proteins - iron regulatory proteins 1 and 2 (IRPs) [9], [10]. IRPs bind to *cis*-regulatory hairpin structures called iron responsive elements (IREs) in the 5′ untranslated regions (UTRs) of target mRNAs and decrease their rate of translation (*Slc40a1*, *Ferritin, HIF-2α*) or in 3′ UTRs to promote mRNA stability (*Slc11a2, transferrin receptor 1*). IRP stability is inversely correlated with the cellular labile iron pool (LIP) [10].

Systemic regulation of iron absorption is achieved through hepcidin (encoded by *HAMP*), a liver-derived iron regulatory hormone sensitive to serum iron, iron stores and erythroid signals [11]. Hepcidin limits the amount of iron released from the duodenum by interacting with FPN at the basolateral membrane and modulating its activity [12], [13]. Regarding copper regulation, an as yet uncharacterized ‘positively acting diffusible signal’ has been shown to promote intestinal and liver copper efflux through its action on ATP7A as a function of systemic/peripheral copper requirements [14].

Perturbations of copper homeostasis leading to imbalances in iron homeostasis are well documented [15]. The iron-copper relationship has focused mainly on HEPH and its homologue ceruloplasmin, (CP), whose half-life, biosynthesis and activity are positively correlated with intracellular copper levels and are required for efficient iron export and loading of iron onto transferrin [16]. Hence copper deficiency, by inhibition of HEPH and CP, alters iron metabolism and restricts iron export from the gut and liver [17], [18]. In addition, a requirement of copper *per se* for heme synthesis has also been demonstrated [19].

Additional local or systemic copper dependent pathways may also affect iron absorption. We hypothesized that changes in either systemic or intestinal copper status regulate HIF-2α and as consequence iron absorption genes since: (i) we and others have shown that *Cybrd1*, *Slc11a2* and *Slc40a1* are *bona fide* Hif-2α targets [6], [7]; (ii) *in vitro* evidence suggests that changes in cellular copper levels regulate HIF-1α [20] and (iii) anemia associated with genetic disorders of copper metabolism could lead to increases in tissue hypoxia and stabilize HIF-2α [21]–[25]. We demonstrate in a mouse model of nutritional copper deficiency an increase in duodenal hypoxia and Hif-2α; i.p. copper injection alleviated copper deficiency anemia, reduced tissue hypoxia and modulated HIF-2α and iron absorption genes (*Slc40a1*, *Slc11a2*, and *Cybrd1*) independently of intestinal iron levels or liver hepcidin. These results provide new insights into how deficit in overall body copper status modulates duodenal hypoxia, HIF-2α and iron absorption genes.

## Materials and Methods

### Animals

Animal studies described here were reviewed and approved (Agreement n° P2.CP.151.10.) by the “Président du Comité d'Ethique pour l'Expérimentation Animale Paris Descartes”. In all experiments both male and female C57BL/6 mice were used. To induce dietary copper deficiency a diet containing between 0.3 and 0.7 ppm of cupric carbonate was used (TD80388, Harlan). Control animals were kept on a control diet (TD.94096), containing approximately 10 ppm of cupric carbonate. To ensure severe copper deficiency, the feeding regiment was modelled on a previously used postnatal (lactational) paradigm [26]. Mice were placed on a copper deficient diet at P1, weaned onto the corresponding diets at P24, and sacrificed at P45. Generation of *Hif-2α* intestinal knockout mice was described previously [7].

### Hypoxyprobe-1 Injections

Mice were injected intraperitoneally (i.p) with a solution of pimonidazole - Hypoxyprobe-1 (60 mg/kg) (NPI, Burlington, USA) diluted in PBS, one hour pre-sacrifice. We dissected 0.5 cm of most proximal part of duodenum and extracted the protein using standard RIPA buffer. We ran 80 µg of protein in a 5% Tris-SDS gel for only 15 min to ensure that the overall protein sample is represented as one band. This approach is different to using resolving gradient gels [27], [28] but does provide for a more accurate quantification of the thiol adducts signal thereafter. Modified thiol adducts were detected with rabbit anti-sera, raised against thiol adducts, provided in the kit. To normalize for loading, we stripped the membrane with Restore Western Blot Stripping Buffer, 21059 (Thermo Scientific) to ensure no residual signal was still present and then re-probed the membrane with mouse β-actin. Proteins were visualized using Image Quant Las4000 mini (GE Healthcare) and quantified with ImageJ (http://rsbweb.nih.gov/ij/).

### Copper Sulphate Intraperitoneal Injections

We injected copper deficient (CuD) mice (P40) with saline or copper sulphate (10 µg/g of body weight) i.p as previously described [29]. Mice were sacrificed after five days (P45).

### Hematological Parameters, Tissue and Plasma Metal Measurements

Copper and iron measurements were performed using Electrothermal Atomic Absorption Spectrometry (ETAAS) on a SIMAA 6100 spectrometer (Perkin Elmer, Courtaboeuf, France). For duodenal measurements, a strip of proximal duodenum (2–3 mm in diameter) was used. Briefly, tissues were digested with nitric acid (14 M). After complete digestion, an appropriate volume of MilliQ water was added to obtain a final dry-tissue concentration of 10 g/L. Plasma samples were directly diluted in 0.1 M HNO_3_ for Cu measurement and pretreated with HCl (1 M) before Fe determination. External calibrations for each metal were performed using four solutions containing increasing amounts of Cu or Fe. Hematological parameters were measured on a MS9-5V apparatus (Melet Schloesing Laboratories).

### Real-time PCR

RNA from liver and duodenum (strip of most proximal duodenum, 2–3 mm in diameter) was extracted using RNA tissue midi Kit (Quiagen). cDNA was prepared using High Capacity cDNA RT (Applied Biosciences) and Real-time PCR was performed using SYBR green on a LC480 Light Cycler (Roche). Primers used are outlined in Table S1. mRNA expression was calculated using the ΔΔCt method and normalized to the expression of *RPLP0*. We have also analysed *HPRT* as an additional housekeeping gene and its expression was not affected by experimental treatments.

### Immunohistochemistry

Immunohistochemical detection of thiol adducts in mouse duodenum was performed using immunoperoxidase detection. Formalin-fixed paraffin embedded sections were processed in a pressure cooker for 5 min at 125°C, blocked with 3% H_2_O_2_ in methanol and 10% goat serum (DAKO, X0907), and incubated with rabbit anti-Hypoxyprobe primary antibody (1∶500, PAb2627) overnight at 4°C. Bound primary antibody was detected by sequentially incubating the samples with goat anti-rabbit biotinylated IgG’s (1∶400, DAKO, E0432) and streptavidin/horseradish peroxidase (1∶400, DAKO, P0397) for 30 min at RT. Chromogen AEC was then added for color development (DAKO, K3464). Images were taken with a light microscope using a 60x resolution objective (Zeiss-Axiophot).

### Western Blotting

Isolated enterocytes were processed and protein fractions isolated as follows: proximal duodenum (2 cm) was washed with ice cold PBS, cut vertically, placed in a 1.5 mM EDTA PBS buffer supplemented with PMSF (500 µM) and protease inhibitors (Roche) and left to shake for 30 min at 4°C. The remaining duodenal muscle was removed and detached enterocytes briefly spun down. The cells were treated with 200 µl of lysis buffer (0.25 M sucrose, 0.03 M L-Histidine, pH = 7.2, 500 µM PMSF and protease inhibitors) for 30 min and intermittently homogenized. The samples were then spun down at 5000 rpm for 10 min at 4°C. The supernatant was removed, spun at 41000 rpm for 1 hr at 4°C to obtain the cytosolic fraction (supernatant) and membrane fraction (pellet resuspended in 15–30 µl of lysis buffer). For the nuclear fraction, the remaining cell pellet was resuspended in a high salt buffer, [30] incubated for 30 min, followed by a spin at 14000 rpm for 10 min at 4°C. Immunoblotting was performed using the following antibodies: HIF-2α (NB100–122) Novus Biologicals; Dcytb (DCYTB11-A, Alpha Diagnostics), DMT1 [31], [32], Hsc70 (sc-7298), CCS (sc-20141), Ferritin H (sc-14416) Santa Cruz and β-actin (A5316) Sigma. Protein concentrations were determined by BCA assay (Thermo Scientific). Proteins were visualized using Image Quant Las4000 mini (GE Healthcare) and quantified with ImageJ.

### Statistical Analysis

Data were analysed in Graphpad Prism 5 using 1-way or repeated measures ANOVA with Newman-Keuls posthoc testing. Correlations were measured using the Spearman coefficient. Data are presented as means±SEM. Where applicable, means without a common letter or symbol are statistically different (*p*<.05).

## Results

### Copper Deficiency Leads to Anemia in Mice

**Figure 1:**
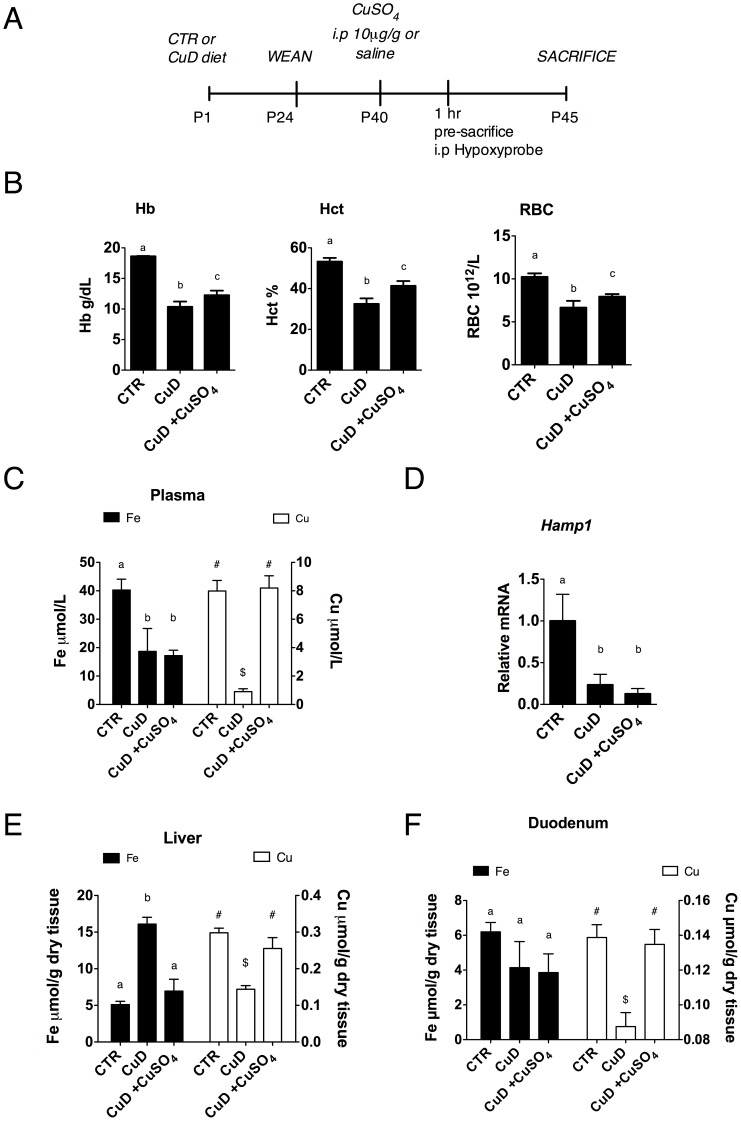
Copper deficiency leads to anemia in mice. (A) Postnatal CuD regime and i.p copper repletion experimental design. (B) Hematological indices of mice at P45 post i.p injection of copper. (C) Plasma iron and copper concentrations. (D) Liver *Hamp1* mRNA levels. mRNA values were adjusted to 1. (E) Liver iron and copper concentrations. (F) Duodenal iron and copper concentrations. 1-way ANOVA with Newman-Keuls posthoc testing. Data are presented as means±SEM: CTR (n = 6), CuD (n≥4), CuD +CuSO_4_ (n≥4). Means without a common letter or symbol differ (*p*<.05).

To determine the extent to which copper deficiency affects duodenal iron homeostasis, an accepted nutritional paradigm of copper deprivation was used. Mice at birth were subjected to a copper deficiency (CuD) regime, weaned at P24 and maintained on their respective diets until P45 (Figure 1A), to ensure that the mice were severely copper deficient. The copper deficient mice at P45 were anemic with decreased hemoglobin (Hb), hematocrit (Hct) and red blood cell count (RBC) (Figure 1B), along with elevated serum erythropoietin levels, weight loss and cardiomegaly (data not shown). Iron and copper content was measured in the proximal duodenum, plasma and liver using atomic absorption spectrometry. Both copper and iron levels were significantly decreased in plasma from CuD mice (Figure 1C), including transferrin saturation (Figure S1A). In accordance, liver *Hamp1* mRNA levels were also decreased in CuD mice relative to controls (Figure 1D) in spite of iron retention in the liver (Figure 1E). Enterocytes from CuD mice were copper deficient but iron was not significantly different when compared to control mice (Figure 1F).

### Hypoxia Regulates Hif-2α Expression and Hif-2α-related Genes in the Duodenum of Copper Deficient Mice

**Figure 2:**
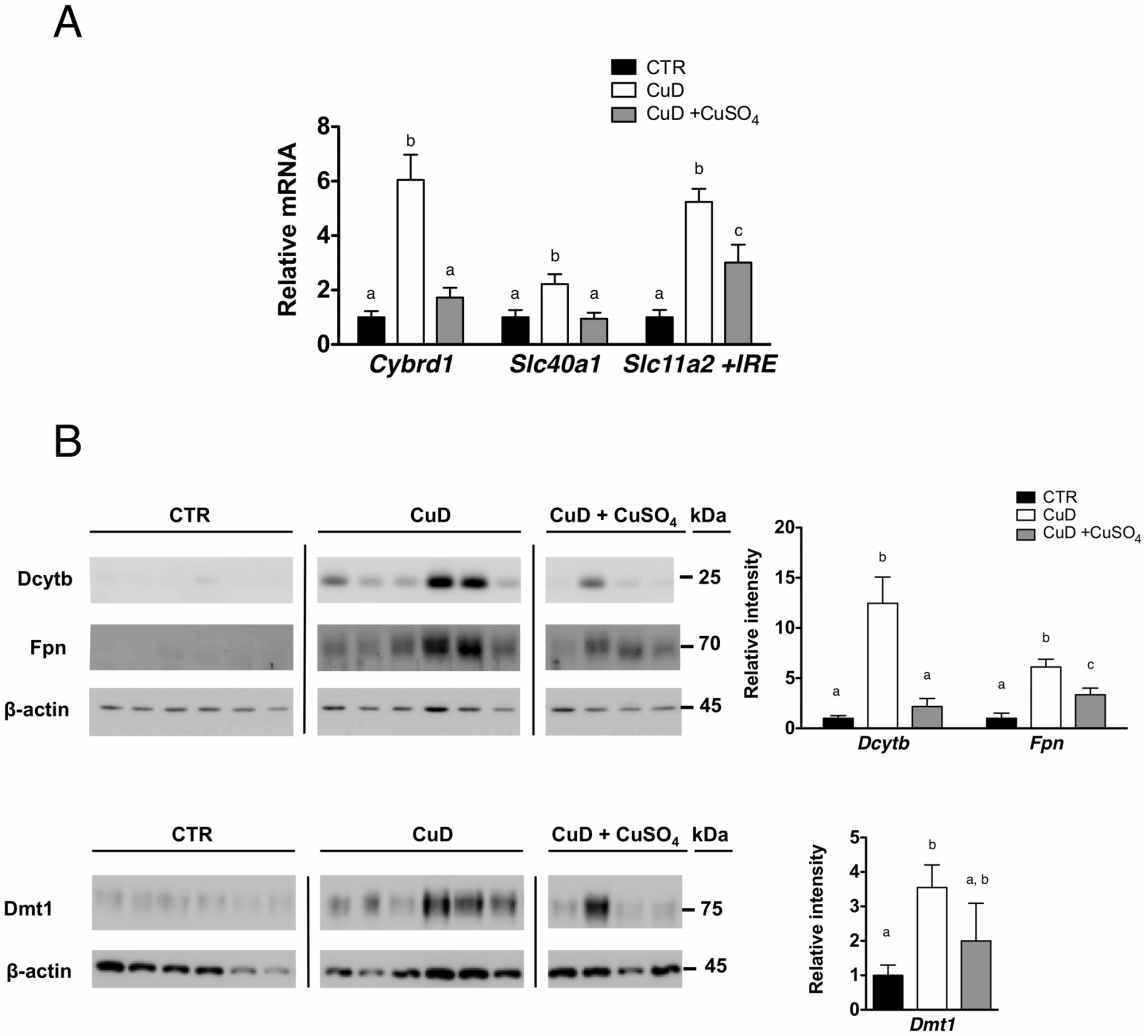
Iron absorption genes are regulated in copper deficiency. (A) Relative mRNA levels of *Cybrd1*, *Slc40a1* and *Slc11a2 +IRE* mRNA in the duodenum of mice on control or CuD diets. mRNA levels of control mice were adjusted to 1. (B) Immunoblots of Dmt1, Fpn and Dcytb (membrane fraction). β-actin was used as a loading control. Changes in protein levels were quantified using ImageJ software. 1-way ANOVA with Newman-Keuls posthoc testing. Data are presented as means±SEM: CTR (n = 6), CuD (n = 6), CuD+CuSO_4_ (n = 4). Means without a common letter differ (*p*<.05).

We next determined the effect of copper deficiency on the expression of iron-related molecules in the duodenum – iron transporters Dmt1 and Fpn and ferric reducatse Dcytb. We found an increase in *Cybrd1*, *Slc40a1* and *Slc11a2 +IRE* mRNA levels in CuD mice as compared to control mice and accordingly an increase in the protein levels as shown in Figures 2A and 2B.

**Figure 3:**
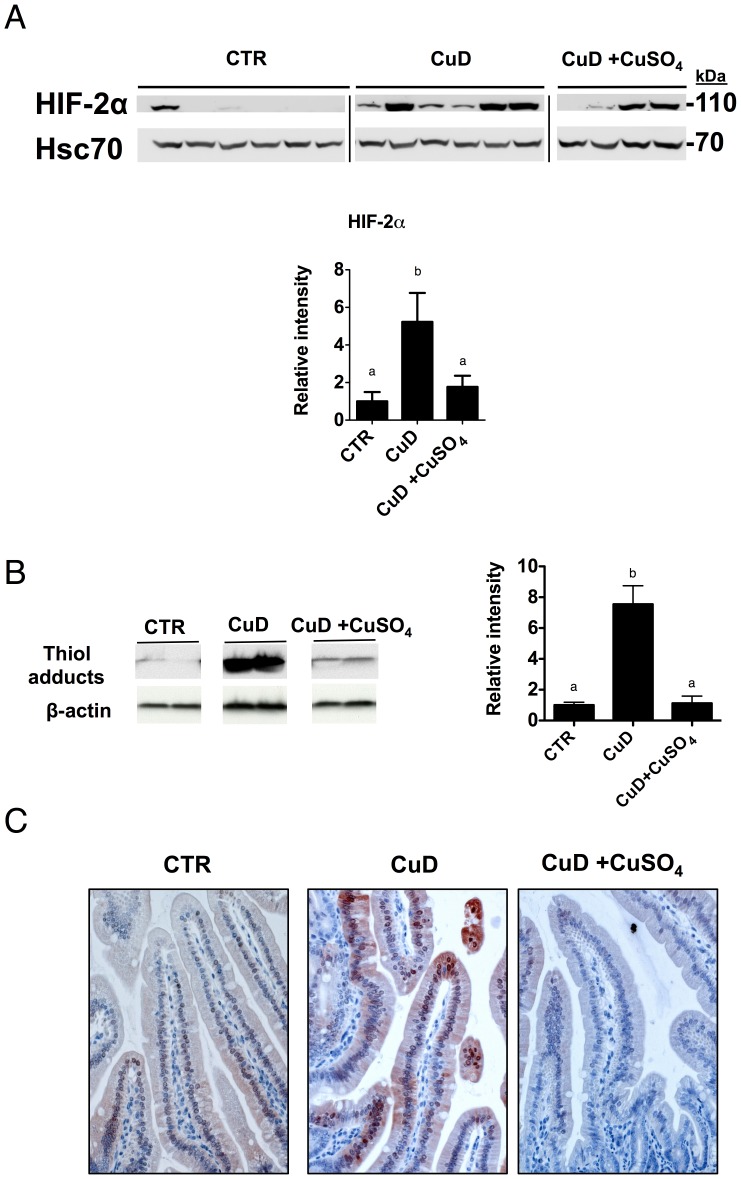
Copper deficiency anemia results in increases in duodenal hypoxia and Hif-2α but partial alleviation of anemia, by copper injection, reduces duodenal hypoxia and down regulates Hif-2α. (A) Immunoblot of Hif-2α (nuclear fraction). Hsc70 was used as a loading control. 1-way ANOVA with Newman-Keuls posthoc testing. CTR (n = 6), CuD (n = 6), CuD+CuSO_4_) (n = 4). (B) Representative immunoblot of thiol adducts (total protein). β-actin was used as a loading control. Band intensity was quantified from 4 samples per group. Repeated measures ANOVA with Newman-Keuls posthoc testing. Data are presented as means±SEM. Means without a common letter differ (*p*<.05). (C) Representative image of thiol adducts in mouse duodenum in the three experimental groups. Immunohistochemical detection of thiol adducts in mouse duodenal villi using Hypoxyprobe™-1 kit immunoperoxidase detection. Chromogen AEC was added for color development.

**Figure 4:**
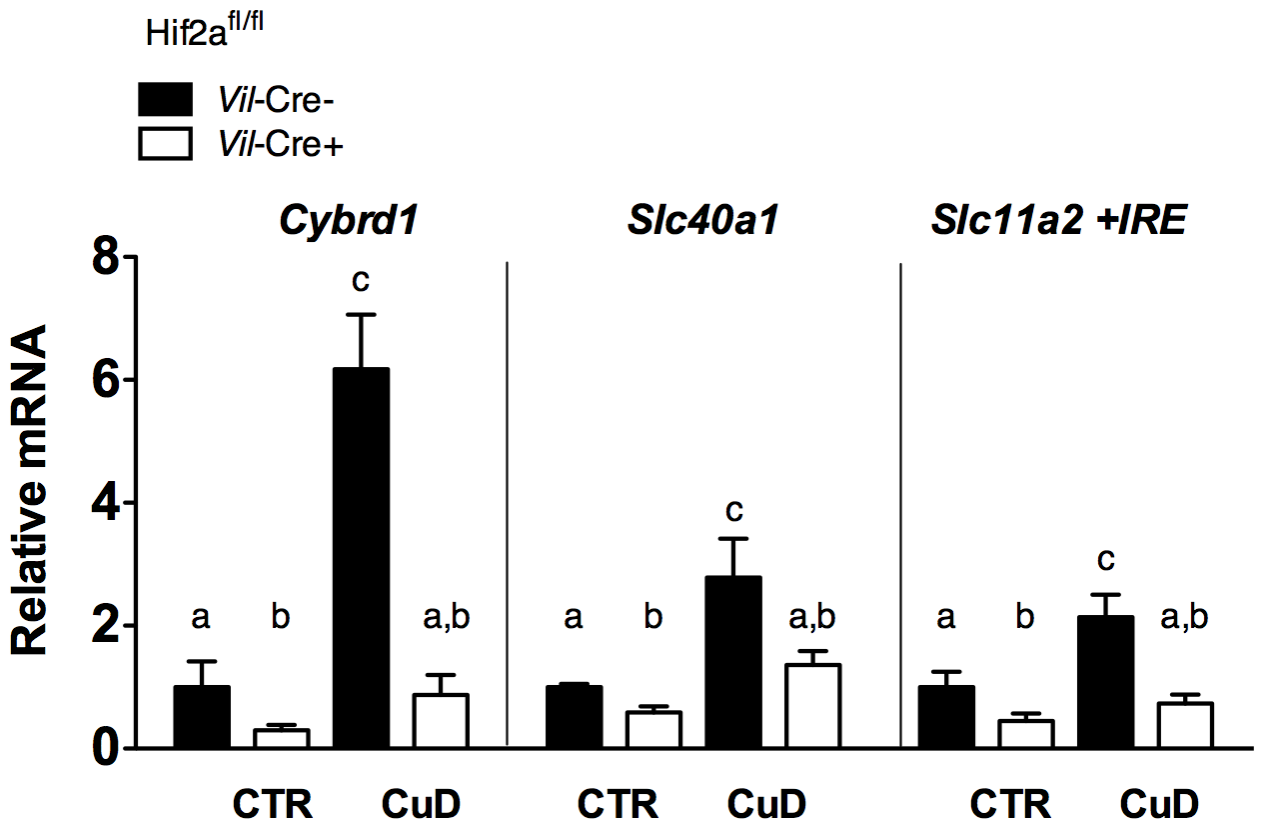
Iron absorption genes are regulated in a HIF-2 dependent manner in copper deficiency. Relative mRNA levels of *Cybrd1*, *Slc40a1* and *Slc11a2 +IRE* from the duodenum of control or CuD *Hif2a* ^fl/fl^ *Vilin*-cre- and *Villin*-cre+ mice. mRNA levels of *Hif2a* ^fl/fl^ *Vilin*-cre- mice on control diet were adjusted to 1. 1-way ANOVA with Newman-Keuls posthoc testing. Data are presented as means±SEM: Control diet - *Hif2a* ^fl/fl^ *Vilin*-cre- (n = 5), *Hif2a* ^fl/fl^ *Villin*-cre+ (n = 7), CuD - *Hif2a* ^fl/fl^ *Vilin*-cre- (n = 5), *Hif2a* ^fl/fl^ *Villin-*cre+ (n = 7). Means without a common letter or symbol differ (*p*<.05).

Since *Cybrd1*, *Slc40a1* and *Slc11a2* are *bona fide* HIF-2α target genes [7], we measured Hif-2α protein levels in the CuD group. We observed an increase in Hif-2α protein level relative to those in control mice (Figure 3A) (with no changes in mRNA levels, data not shown) demonstrating for the first time that Hif-2α was upregulated in the duodenum in copper deficiency anemia and that it correlated with increases in *Cybrd1* (r = 0.7439, p<.0001), *Slc40a1* (r = 0.686, p<.001) and *Slc11a2 +IRE* (r = 0.6439, p<.001) mRNA levels. Moreover, to confirm this correlation, we subjected *Hif-2α* intestinal knockout mice to a copper deficient diet [7]. The increased expression levels of all three genes in *Hif2a* ^fl/fl^ *Vilin*-cre+ mice were significantly attenuated relative to the control *Hif2a* ^fl/fl^ *Vilin*-cre- mice (Figure 4). Since HIF-2α is acutely stabilized by hypoxia in the duodenum [33], we next quantified the degree of tissue hypoxia in the CuD mice by injecting pimonidazole hypoxyprobe one hour pre-sacrifice, and detected the amount of thiol adducts. We found a significant increase in thiol protein adducts in CuD mice, as shown by immunoblotting (Figure 3B) and by immunochemistry of the duodenal villi (Figure 3C). Therefore, copper deficiency leads to anemia, increases in duodenal hypoxia, HIF-2α and iron absorption genes.

### Intraperitoneal Copper Injection Alleviates Anemia, Reduces Gut Hypoxia and Down Regulates Hif-2α and Iron Absorption Genes

To establish if anemia/hypoxia is the dominant signal, which affects duodenal HIF-2α levels and HIF-2α-regulated iron genes, we performed i.p. copper repletion experiments. Mice were injected at P40 with either 10 µg/g body weight CuSO_4_ i.p. or saline and sacrificed five days post injection, as described (Figure 1A) [29]. We observed, post-copper injection, a significant amelioration of Hb, Hct and RBC counts (Figure 1B) indicative of increases in erythropoiesis. In addition, we also measured marked increase in spleen size in CuD, copper treated mice indicative of enhanced extramedullary erythropoiesis (Figure S1B). These changes were concomitant with iron mobilization from the liver (Figure 1E). Furthermore, plasma and duodenal copper levels, five days post injection, returned to control levels while their respective iron content remained unchanged (Figures 1C, 1F). Hepcidin levels were not significantly different post copper injection (Figure 1D).

As anticipated, we found, in the duodenum, that the increases of Hif-2α protein and hypoxyprobe levels were attenuated in the CuD mice injected with copper (Figures 3A–C). Accordingly, the HIF-2α target genes *Cybrd1, Slc40a1* and *Slc11a2 +IRE,* paralleled the expression of Hif-2α, with Dcytb and Fpn being significantly decreased after copper injection (Figures 2A and 2B). Together, these results demonstrate that partial correction of anemia, by i.p copper injection, as exampled in these sets of experiments, down regulated Hif-2α and Hif-2α target genes, independently of duodenal iron or liver hepcidin.

## Discussion

The data presented demonstrate a new relationship between copper and iron homeostasis. We show an increase in duodenal hypoxia in a nutritional model of copper deficiency anemia, and Hif-2α upregulation. This increase appears to be independent of local iron content. By injecting copper parenterally to rescue the anemia, we demonstrated down regulation of iron absorption genes and Hif-2α. We were interested in elucidating the copper-dependent pathways, which could stabilize HIF-2α and regulate iron absorption genes in copper deficiency. We reasoned that either copper deficit *per se*, changes in cellular iron levels, or increases in tissue hypoxia might mediate these events. We conclude that tissue hypoxia is the principal driving force for the copper-dependent pathway that upregulates Hif-2α and regulates iron absorption genes in copper deficiency in mice.

To our knowledge, there are no data available demonstrating regulation of HIF-2α by copper, although there is some *in vitro* evidence for HIF-1α. Martin *et al.* have shown that copper repletion stabilizes HIF-1α in normoxia through the inhibition of PHDs [20], whereas Jiang *et al.* showed copper supplementation promotes HIF-1α gene *trans*-activation in the heart [34]. Conversely, the same authors showed that copper chelation inhibited HIF-1α binding and *trans*-activation of target promoters [35]. *In vitro* results from our laboratory indicate that copper chelation in cell culture does not stabilize HIF-2α protein or promote HIF-2α dependent gene *trans*-activation (Figure S3). This suggests that mechanisms other than cellular copper deficiency regulate HIF-2α in our model.

The cellular iron content could also influence HIF-2α activity. Iron loading can lead to de-repression of HIF-2α translation via IRP1 through a 5′ UTR IRE in the *HIF-2α* transcript [9], [36]. However, in our nutritional copper deficiency model (Figures 1, 2, 3), duodenal iron content was not significantly altered relative to the control levels, yet Hif-2α was significantly increased. Indeed a number of studies, across species, have shown that iron retention in the gut, (associated with copper deficiency due to a significant decrease in hephaestin feroxidase activity and thus iron export) is not always observed in copper deficiency [17], [37]–[39] and this provides evidence that cellular iron levels *per se* may not be the major determinant of HIF-2α protein levels.

Deficit of either copper or iron is known to lead to impairment of hemoglobin synthesis and consequentially reduce the oxygen carrying capacity of erythrocytes, resulting in systemic tissue hypoxia. Our hypoxyprobe and copper repletion experiments demonstrate that HIF-2α upregulation in copper deficiency is driven by systemic hypoxia. In support of this finding, recent data have shown that severe phenylhydrazine-induced hemolysis resulted in systemic tissue hypoxia and Hif-2α upregulation in the duodenum [27].

Hif-2α upregulation in copper deficiency correlated with regulation of iron absorption-related genes, and we further confirmed this correlation directly in *Hif-2α* intestinal knockout mice under copper deficient diet in a separate experiment (Figure 4) [7]. Increases in mRNA levels for all three transcripts were attenuated in copper deficient *Hif2a* ^fl/fl^ *Vilin*-cre+ mice relative to the control *Vilin*-cre- mice. Thus iron absorption genes are upregulated in copper deficiency in a Hif-2α dependent manner. We propose this to be a physiologically relevant mechanism to adjust iron absorption relative to the degree of anemia/erythropoietic impairment resulting from changes in systemic copper status. This would involve at least 3 steps:

1. Upregulation of FPN as a compensatory response to systemic copper deficit to adjust the rates of iron export. This in turn is likely linked to decreased activity of the copper-dependent oxidases HEPH and CP. In support of this mechanism, Chen *et al.* have shown an increase in *Slc40a1* mRNA levels in sex-linked anemia (sla) mice that carry an in-frame deletion in the *Heph* gene, resulting in partial loss of its activity and systemic iron deficiency [40], [41]. In a agreement with our study increased duodenal *Slc40a1* mRNA levels have also been reported in CuD mice [26]. Although we have not measured hephaestin activity directly it is likely to be significantly reduced. However other intestinal ferroxidases are known to be present in the gut, which could compensate, in part, for the loss of hephaestin and maintain some degree of iron efflux function [42], [43]. Furthermore, *Hamp1* levels in our copper-deficiency model were decreased, in agreement with a previous study [26]. *Hamp1* levels were also not changed post copper injection although Fpn protein levels decreased, suggesting the latter to be a hepcidin independent event.
2. Modulation of apical Dmt1 expression to regulate iron absorption. The *Slc11a2 +IRE* transcript isoform predominates in the duodenum and its levels are thought to be dependent on at least two opposing cues in copper deficiency: systemic hypoxia regulation, via HIF-2α-mediated *trans*-activation of the *1a* promoter and local regulation, via IRP-mediated effects on mRNA stability (although the latter has not been formally demonstrated *in vivo*). Based on our current data, we propose that the increase in *Slc11a2 +IRE* levels in copper deficiency is due to a dominant effect of HIF-2α (no iron retention but significant duodenal hypoxia). In support of this hypothesis, post-copper rescue, there was no significant change in enterocyte iron levels compared with CuD mice, but *Slc11a2 +IRE* and HIF-2α levels decreased and there was a reduction in duodenal tissue hypoxia. However, severe copper deficiency in a number of animal studies has been shown to lead to iron retention in the gut due to impairment in basolateral iron export [26], [44]–[46]. As such, high iron levels are expected to have a negative effect on Dmt1 expression. Indeed, in a separate set of experiments, when we submitted mice to a prenatal copper deficiency regime (E17 through to P38), which resulted in significant iron retention in the gut, we observed no differences in Dmt1 +IRE expression. This was in contrast to Fpn, Dcytb and Hif-2α which all increased (Figure S2). Thus the balance between systemic (hypoxia) and local (iron) cues is a major determinant of *Slc11a2 +IRE* expression under copper deficiency, however additional layer/s of regulation cannot be ruled out, for example the effects of hypoxia on IRP activity [47].
3. Dcytb upregulation in copper deficiency to promote iron and copper absorption. *Cybrd1* is one of a number of iron-related genes not to contain an IRE sequence. The lack of a 3′ UTR IRE, as found in *Slc11a2,* therefore allows *Cybrd1* levels to be up-regulated via HIF-2α under both copper and iron deficiency. Recent studies demonstrate that both Dcytb and the Steap proteins (2–4) are metalloreductases [2], [3]. In the case of the Steap proteins, over-expression results in increased copper uptake. More importantly however, in copper deficiency, we found *Cybrd1*, but not the *Steaps* (data not shown) to be significantly upregulated in the duodenum. It is tempting to envisage, depending on the context, Dcytb working in tandem with Ctr1 to enhance copper uptake. Thus the role of Dcytb or Steaps in copper uptake *in vivo* warrants further analysis, for example using respective mouse knockout models.

**Figure 5:**
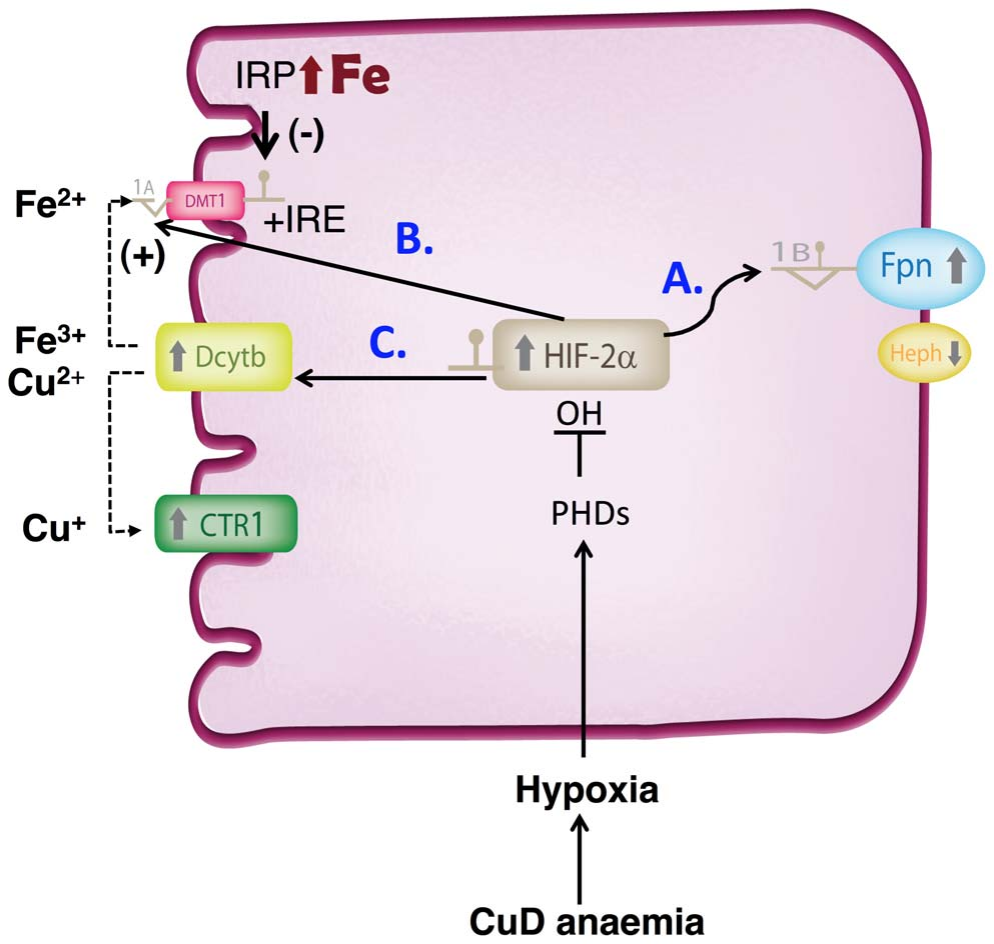
Model for the role of HIF-2α in the adaptive response to copper deficiency anemia. HIF-2α upregulation results in altered expression of iron absorption genes as a result of systemic copper deficit/anemia. This leads to: a) increases in Fpn levels to compensate/adjust the rates of iron export to systemic copper decreases; b) systemic modulation of *DMT1+IRE* expression (HIF-2α *trans*-activation of the *DMT1-1a* promoter). The net expression of DMT1 is likely influenced by the amount of iron retained in the gut (associated with CuD due to a significant decrease in hephaestin feroxidase activity), and the effect on IRP activity. On one hand, in mild or moderate CuD (no iron retention) systemic HIF-2α increases are likely to exert an overall dominant effect on *DMT1* expression (Figure 2). At the other extreme, in severe CuD resulting in enterocyte iron loading, decreases in IRP stability and thus *Dmt1+IRE* mRNA half-life, would counterbalance the HIF-2α transcriptional upregulation of DMT1, thus decreasing, or not altering *DMT1* expression (Figure S2); c) increases of Dcytb in CuD, apart from iron uptake and depending on context, could also promote copper uptake in tandem with Ctr1.

In summary, we report a new paradigm for modulating iron absorption through hypoxia/HIF-2, independent of hepcidin and duodenal iron levels, but reflective of overall body copper status (Figure 5). Copper-iron interactions regulating intestinal iron absorption have been clearly defined in recent years [4], [48]. Our work extends this body of evidence and identifies HIF-2α as a regulator of the intestinal iron transport machinery in copper deficiency.

## Supporting Information

Figure S1 Additional systemic parameters. (A) Plasma transferrin saturation, (B) spleen size (% body weight) 1-way ANOVA with Newman-Keuls posthoc testing. Data are presented as means±SEM: CTR (n = 6), CuD (n = 5), CuD+CuSO_4_ (n≥4). Means without a common letter differ (*p*<.05).
(TIFF)

Figure S2 Prenatal nutritional copper deficiency regime - HIF-2α, Dcytb and Fpn upregulation but no change in Dmt1+IRE in copper deficient but iron loaded duodenum. (A) Experimental scheme of the prenatal copper deficiency regime. To test whether iron loading may affect the expression of HIF-2α and iron related genes in the context of copper deficiency, we submitted mice to a prenatal copper deficiency regime. C57BL/6 pregnant dams (E14) were purchased from Harlan (Gannat, France) and placed on a CuD or copper adequate diet at E17. Pups were weaned at P24 and maintained on their respective diets until P38. (B) Hematological indices of control (CTR) and copper deficient (CuD) mice at P37. Unpaired student t-test (***p<.001); CTR (n = 15), CuD (n = 8). (C) Duodenal iron and copper concentrations were measured by atomic absorption spectroscopy. Iron in the proximal duodenum was significantly increased, in contrast to copper, which decreased. Unpaired student t-test (***p<.001); CTR (n = 15), CuD (n = 8). (D) Relative mRNA levels of *Cybrd1*, *Slc40a1* and *Slc11a2 +IRE* mRNA in the duodenum of mice on control or copper deficient diets. mRNA levels of control mice were adjusted to 1. Unpaired student t-test (*p<.05, ***p<.001); CTR (n = 5), CuD (n = 5). (E) Representative immunoblots (membrane fraction) of Dmt1+IRE (NRAMP21-A, Alpha Diagnostics), Fpn, Dcytb, Hif-2α (nuclear fraction) and Ferritin H (cytosolic fraction). β-actin and Hsc70 were used as loading controls.
(TIFF)

Figure S3 Effect of copper chelation on HIF-mediated gene *trans*-activation and HIF-2α protein stability. (A) The effect of TETA treatment of HIF-2α protein stability in Caco-2 intestinal cells. Caco-2 cells were treated with incremental doses of TETA for 24 hours. HIF-2α immunoblot (nuclear fraction) with a Ponceau stain as a loading control. (B) HREB cells [20], stably transfected with a hypoxia responsive luciferase reporter were treated with in a copper chelator Triethylenetetramine-TETA (500 µM ) in normoxia (20%) or hypoxia (1%) for 24 hrs. In normoxia, TETA treatment had no effect on reporter activity, whereas in hypoxia a mild but significant reduction in reporter *trans*-activation was observed. 1-way ANOVA with Newman-Keuls posthoc testing. Data are presented as means±SEM. The experiment was performed in triplicate per group. Relative light units (RLU) were measured on a luminometer using a Dual Glo Luciferase Kit (Promega) as previously outlined [7].
(TIFF)

Table S1 Primers sequences used for Real-time PCR.
(EPS)
