# Supplementary figures and images for: Copper Deficiency Leads to Anemia, Duodenal Hypoxia, Upregulation of HIF-2α and Altered Expression of Iron Absorption Genes in Mice
Source: PLoS One. 2013 Mar 28;8(3):e59538. doi: 10.1371/journal.pone.0059538 (PMC3610650; doi:10.1371/journal.pone.0059538)

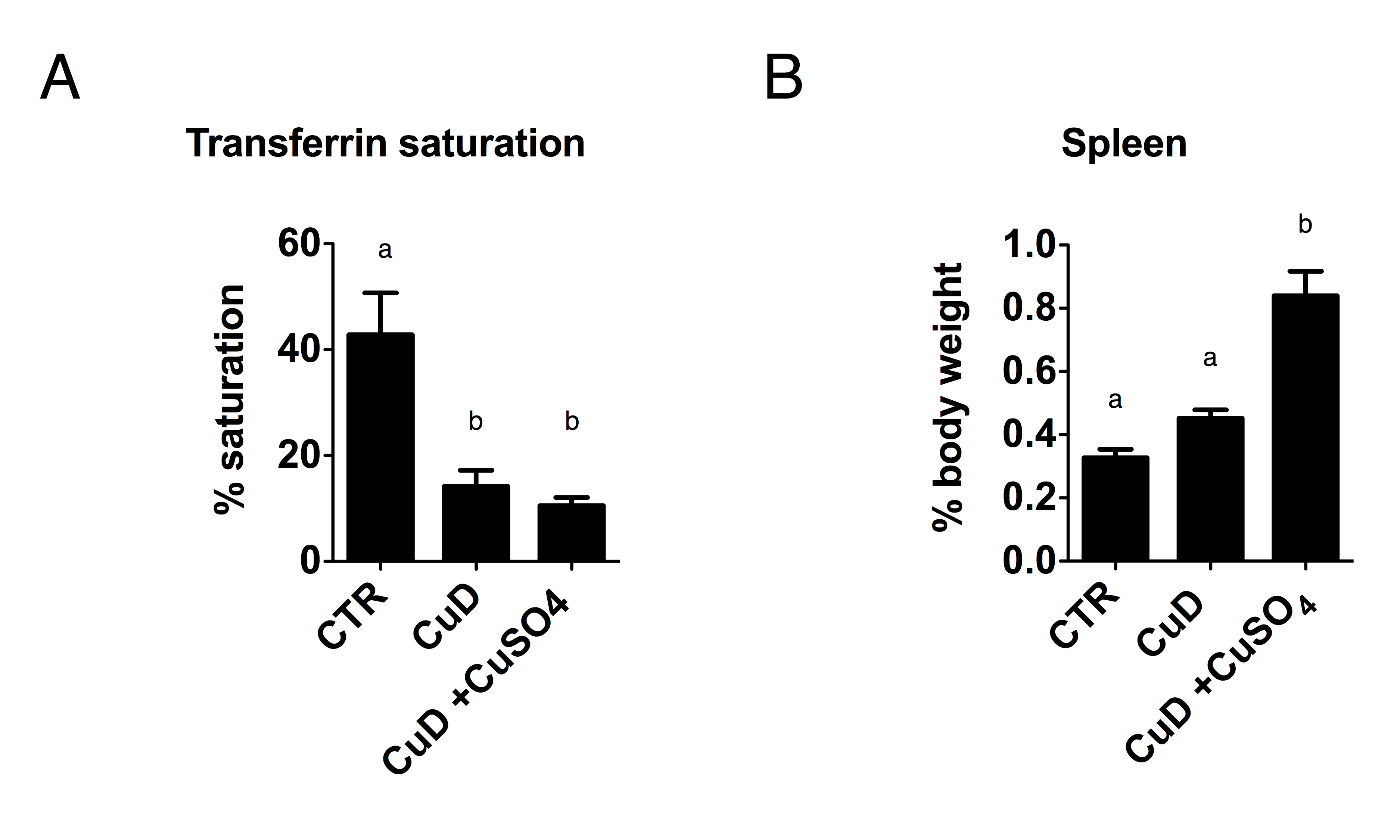

Supplement: Figure S1 — Additional systemic parameters. (A) Plasma transferrin saturation, (B) spleen size (% body weight) 1-way ANOVA with Newman-Keuls posthoc testing. Data are presented as means±SEM: CTR (n = 6), CuD (n = 5), CuD+CuSO4 (n≥4). Means without a common letter differ (p<.05). (TIFF) [file pone.0059538.s001.tiff]

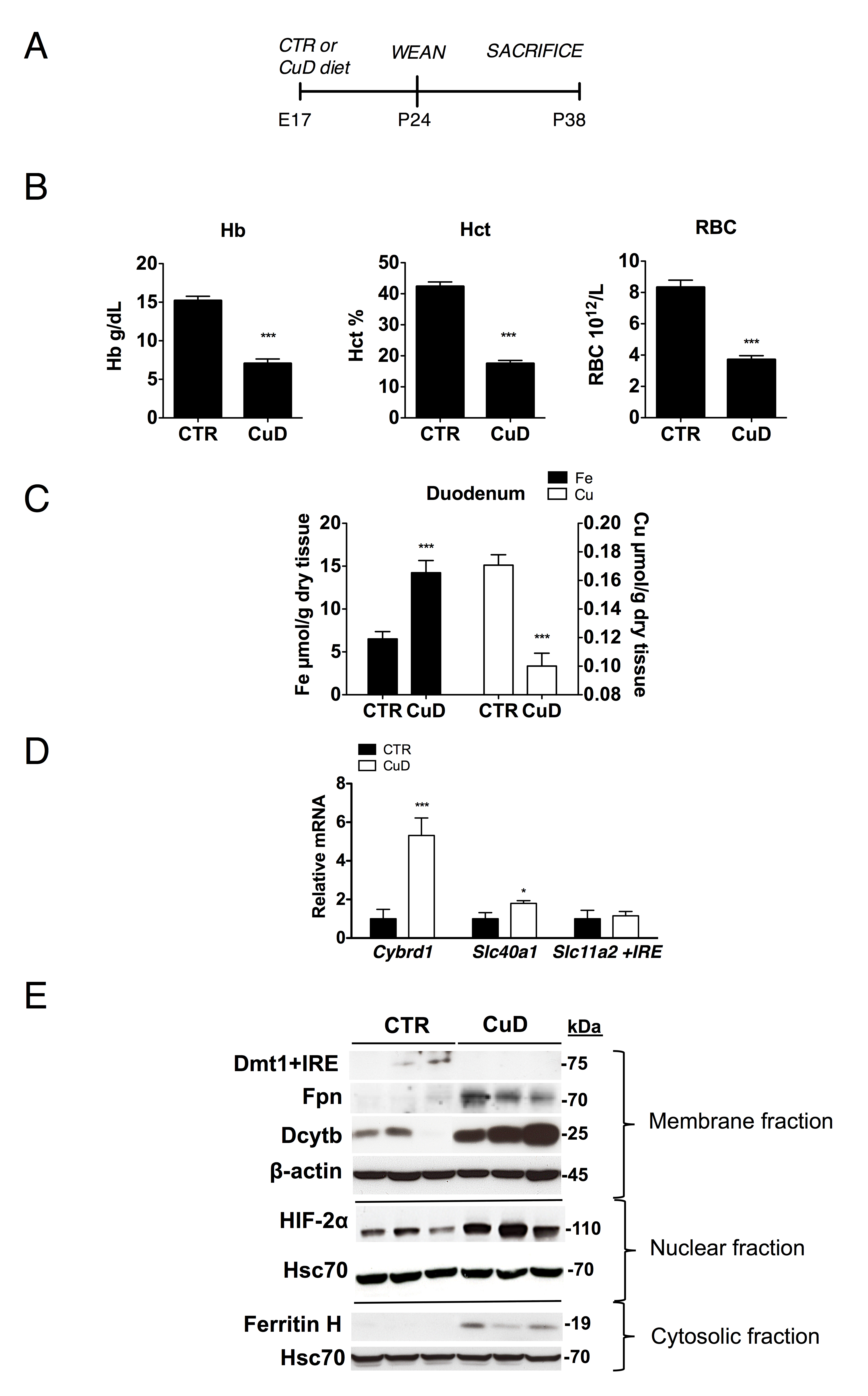

Supplement: Figure S2 — Prenatal nutritional copper deficiency regime - HIF-2α, Dcytb and Fpn upregulation but no change in Dmt1+IRE in copper deficient but iron loaded duodenum. (A) Experimental scheme of the prenatal copper deficiency regime. To test whether iron loading may affect the expression of HIF-2α and iron related genes in the context of copper deficiency, we submitted mice to a prenatal copper deficiency regime. C57BL/6 pregnant dams (E14) were purchased from Harlan (Gannat, France) and placed on a CuD or copper adequate diet at E17. Pups were weaned at P24 and maintained on their respective diets until P38. (B) Hematological indices of control (CTR) and copper deficient (CuD) mice at P37. Unpaired student t-test (***p<.001); CTR (n = 15), CuD (n = 8). (C) Duodenal iron and copper concentrations were measured by atomic absorption spectroscopy. Iron in the proximal duodenum was significantly increased, in contrast to copper, which decreased. Unpaired student t-test (***p<.001); CTR (n = 15), CuD (n = 8). (D) Relative mRNA levels of Cybrd1, Slc40a1 and Slc11a2 +IRE mRNA in the duodenum of mice on control or copper deficient diets. mRNA levels of control mice were adjusted to 1. Unpaired student t-test (*p<.05, ***p<.001); CTR (n = 5), CuD (n = 5). (E) Representative immunoblots (membrane fraction) of Dmt1+IRE (NRAMP21-A, Alpha Diagnostics), Fpn, Dcytb, Hif-2α (nuclear fraction) and Ferritin H (cytosolic fraction). β-actin and Hsc70 were used as loading controls. (TIFF) [file pone.0059538.s002.tiff]

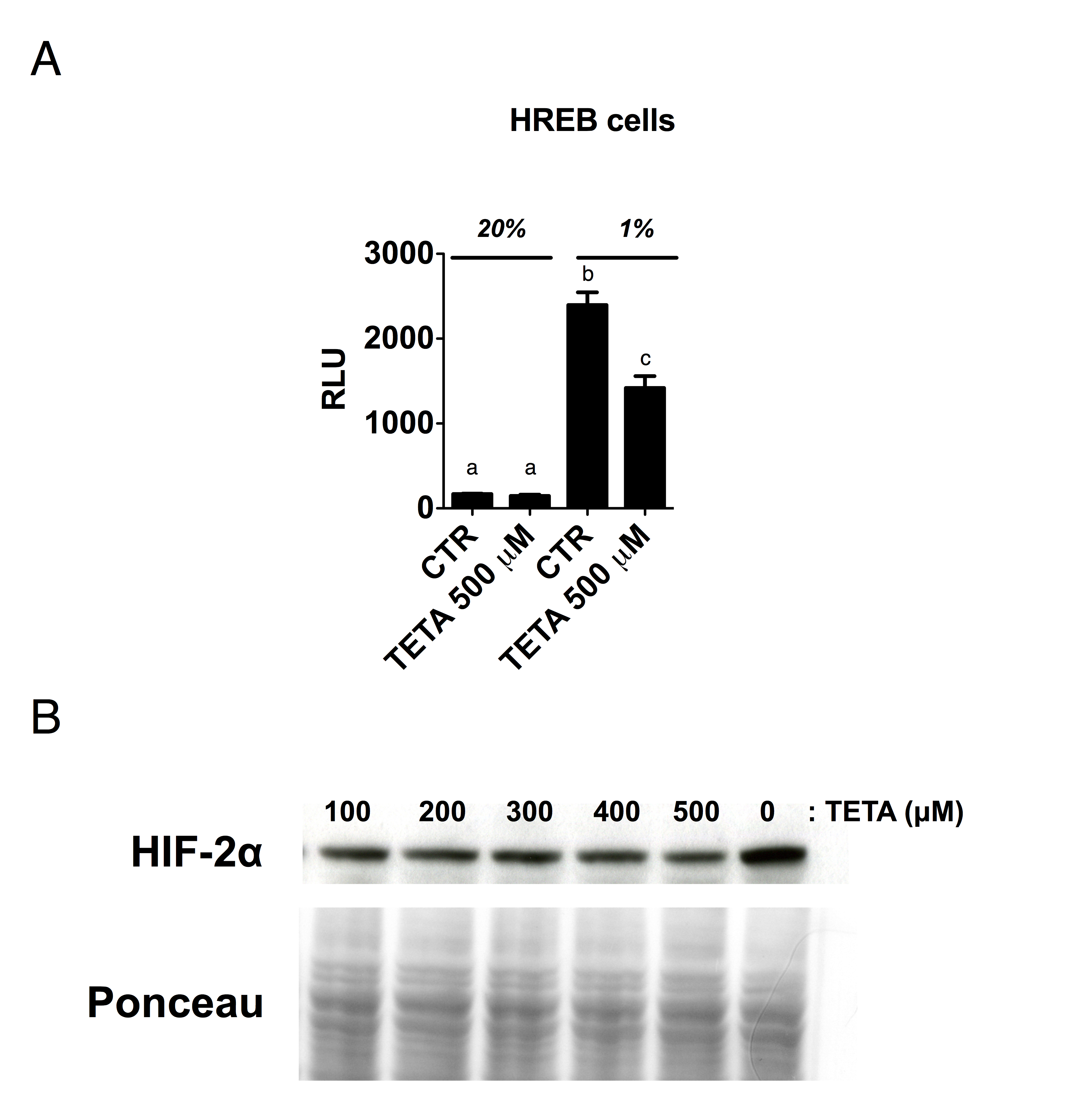

Supplement: Figure S3 — Effect of copper chelation on HIF-mediated gene trans-activation and HIF-2α protein stability. (A) The effect of TETA treatment of HIF-2α protein stability in Caco-2 intestinal cells. Caco-2 cells were treated with incremental doses of TETA for 24 hours. HIF-2α immunoblot (nuclear fraction) with a Ponceau stain as a loading control. (B) HREB cells [20], stably transfected with a hypoxia responsive luciferase reporter were treated with in a copper chelator Triethylenetetramine-TETA (500 µM ) in normoxia (20%) or hypoxia (1%) for 24 hrs. In normoxia, TETA treatment had no effect on reporter activity, whereas in hypoxia a mild but significant reduction in reporter trans-activation was observed. 1-way ANOVA with Newman-Keuls posthoc testing. Data are presented as means±SEM. The experiment was performed in triplicate per group. Relative light units (RLU) were measured on a luminometer using a Dual Glo Luciferase Kit (Promega) as previously outlined [7]. (TIFF) [file pone.0059538.s003.tiff]
